# Excess mortality during COVID-19 and prediction of mortality in Bangladesh: an analysis based on death records in urban graveyards

**DOI:** 10.7189/jogh.15.04050

**Published:** 2025-02-28

**Authors:** Ema Akter, Aniqa Tasnim Hossain, Bibek Ahamed, Md Hafizur Rahman, Tanvir Hossain AKM, Uchchash Barua, Md Shahidul Islam, Ridwana Maher Manna, Md Alamgir Hossain, Tasnu Ara, Nasimul Ghani Usmani, Pradip Chandra, Shafiqul Ameen, Sabrina Jabeen, Anisuddin Ahmed, Taufiq Zahidur Rahman, Mohammad Mamun-Ul-Hassan, Atiqul Islam, Beth Tippett Barr, Qazi Sadeq-ur Rahman, Shams El Arifeen, Ahmed Ehsanur Rahman

**Affiliations:** 1 Maternal and Child Health Division, International Centre for Diarrhoeal Disease Research, Bangladesh (icddr,b), Dhaka, Bangladesh; 2 Dhaka North City Corporation (DNCC), Dhaka, Bangladesh; 3 Nyanja Health Research Institute, Salima, Malawi

## Abstract

**Background:**

The COVID-19 pandemic significantly impacted global mortality, underscoring the need for reliable data to guide public health policy. In low- and middle-income countries, graveyard-based death records can offer valuable insights into COVID-19-related mortality, yet they remain limited. Additionally, data on mortality beyond the pandemic remains scarce as we approach the 2030 Sustainable Development Goals. We addressed this gap by using graveyard-based data to assess excess mortality during the pandemic (2020–23) and predict mortality trends through 2030.

**Methods:**

We analysed 70 585 deaths from six graveyards in Dhaka, Bangladesh, from January 2001 to December 2023. The data was divided into pre-COVID-19 (2001–19), peak-COVID-19 (2020–21), and end-of-COVID-19 (2022–23) phases. We assessed the excess mortality using the P-score and Bayesian approach. We estimated excess mortality with a log-linear Bayesian model and predicted death trends for 2024–30, reporting incidence rate ratio (IRR) with 95% credible intervals (CrI).

**Results:**

Overall, excess mortality was 69% greater in 2020 and 31% in 2023 compared to the 2018–19 average. The IRR for deaths during peak-COVID-19 was 1.66 times higher than pre-COVID-19 (95% CrI = 1.35–2.04). Neonates had significantly higher IRRs during both the peak (IRR = 1.45; 95% CrI = 1.02–2.05) and end-of-COVID-19 (IRR = 1.67; 95% CrI = 1.02–2.71). Individuals aged >40 years showed a significantly higher IRR during peak-COVID-19 (IRR = 1.79; 95% CrI = 1.46–2.18). Predictions using data between 2001–23 indicate rising mortality, with the number of adult deaths increasing from 3318 in 2023 to 5089 (95% CrI = 3871–6267) by 2030.

**Conclusions:**

We revealed a significant rise in mortality during the pandemic, with elevated death rates persisting at the end of the pandemic. Predictions indicate continued mortality increases through 2030, underscoring the pandemic’s long-term health impacts. While further research is needed, these findings highlight the value of graveyard-based death registration data for tracking mortality trends and informing public health strategies.

The COVID-19 pandemic has had a profound impact on people’s lives globally. To understand its true impact, reliable data and accurate measurements across regions and countries are needed [1]. Accurately quantifying COVID-19-related deaths, especially in low- and middle-income countries like Bangladesh, poses significant challenges because of population density, diverse public health measures, and the quality of civil registration and vital statistics (CRVS) systems [2]. Bangladesh has experienced multiple waves of COVID-19 since 2020, prompting the establishment of different mechanisms to track COVID-19 deaths and infections across the country [3]. Official reports indicate approximately 29 500 COVID-19 deaths in Bangladesh [4]. However, World Health Organization (WHO) modelling estimates indicated nearly 141 000 people died due to COVID-19 in Bangladesh between 2020–21 [5], indicating significant underreporting occurred in the country. This discrepancy highlights the inadequacy of existing routine mortality surveillance systems in capturing the pandemic’s actual impact on mortality rates [6].

In Bangladesh, the CRVS suffers from poor quality and incompleteness [7]. The weak CRVS systems provide us with remarkably little insight into the magnitude of excess mortality associated with the COVID-19 pandemic. Hospitals, main sources of cause-specific death data, are not integrated into the CRVS system. Additionally, since the majority of deaths occur at home, many causes of death go unrecorded or are misclassified. Moreover, Bangladesh’s death registration system is still paper-based [8]. These issues likely caused significant delays in death reporting, which led to delays in appropriate responses by policymakers. During the pandemic, many organisations worked on reporting and tracking mortality [9], but their stand-alone systems and lack of collaboration were a missed opportunity to create a complete picture of the true pandemic impact [10].

According to a study conducted in Matlab, a rural area of Bangladesh with a Health and Demographic Surveillance System, the mortality rate per 1000 person-years increased from 10 in 2019 to 12 in 2020, and the elderly mortality rate per 1000 person-years rose from 80 in 2019 to 110 in 2020 [11]. Another urban graveyard-based study in Jamalpur town, northwest of Bangladesh, found more deaths during the COVID-19 pandemic period compared to pre-COVID-19 pandemic [12]. In comparison, one rural Bangladesh survey assessing excess mortality found lower all-cause mortality in 2020 compared to 2019, with death rates lower in rural than urban areas [13], indicating there may have been mortality variation between urban and rural areas during COVID-19.

Graveyard-based death registration presents a potential for an innovative and inexpensive data source in Bangladesh. We designed, implemented, and evaluated a digital mortality surveillance system in graveyards in Dhaka, Bangladesh. We have established the feasibility of digitalising this mortality surveillance. This system can also be used to predict the number of deaths using appropriate statistical modelling. Ensuring a reliable graveyard-based data source can also serve as an early warning system by predicting deaths to track the impact of any future epidemics.

In this study, we aimed to explore the potential of graveyard-based death data to report excess mortality during COVID-19 between 2020–23 and to predict mortality trends up to 2030. To our knowledge, this is the first study utilising historical graveyard records for prediction. In this study, we offer critical insights for public health planning and policymaking, emphasising the need for robust mortality data to inform sustainable health interventions and policy decisions.

## METHODS

### Study setting

We used graveyard-based data extracted from graveyards in Bangladesh, a South Asian country with a diverse population. As per the Population and Housing Census 2022, Bangladesh has a population of approximately 165 million, making it one of the most densely populated countries globally, with 1119 people/m^2^ [14]. Bangladesh is divided into eight divisions (Barishal, Chattogram, Dhaka, Khulna, Rajshahi, Rangpur, Mymensingh, and Sylhet), further divided into 64 districts.

Dhaka, the capital and largest city of Bangladesh, ranks as the sixth most densely populated city in the world [15]. Dhaka City Corporation, established as a self-governing entity for the administration of Dhaka, was split into Dhaka North City Corporation (DNCC) and Dhaka South City Corporation in 2011 for better efficiency at a functional level. We identified six public graveyards under DNCC – Uttara Sector 12, Uttara Sector 14, Uttara Sector 4, Banani Graveyard, Rayer Bazar, and Mirpur Buddhijibi. Among these six graveyards, Banani graveyard, established in 1974, is the oldest, while Uttara Sector 14, established in 2019, is the newest (Figure S1 in the **Online Supplementary Document**). In 2020, we started an exploratory study in these six graveyards managed by DNCC.

### Graveyard-based data

We extracted death records from the graveyard registry after thoroughly understanding the burial site’s basic characteristics, burial practices, and data flow. Our data collection team captured snapshots of every available record using mobile devices and cameras. These records included the deceased’s name, father or husband’s name, age, address, place and cause of death, contact number of a relative, and the deceased’s relationship to the information provider. During the process, we also photographed other supporting documents, such as national identification number, birth certificates, and death certificates. Our data management team then completed data entry from the death registers using a structured format, digitising all death records extracted from DNCC graveyards from the establishment of the burial site until February 2024. We found more than 111 500 death records from 1974 to 2023, with 60 720 coming from Mirpur Buddhijibi graveyard (Figure S1 in the **Online Supplementary Document**).

Of the extracted death records, 102 754 (92.1%) were properly documented. Of these, 32 108 (31.2%) deaths occurred between 1974–2000, and 70 585 (68.7%) occurred between 2001–23 (Figure S2 in the **Online Supplementary Document**). For the analysis in this study, we considered the 70 585 deaths that occurred from January 2001 to December 2023.

### Statistical analysis

We used time series data of monthly death counts between 2001–23. We divided the time period into three distinct phases: pre-COVID-19 (January 2001–December 2019), peak-COVID-19 (January 2020–December 2021), and end-of-COVID-19 (January 2022–December 2023).

To quantify excess mortality, we initially calculated the classical P-score for each graveyard in DNCC and age-specific by comparing the total number of deaths during the COVID-19 period (2020–23) with the immediate previous years (2018–19). The P-score, a measure of excess mortality, was calculated as the percentage difference between the number of deaths in the current period and the average number of deaths in the previous period:



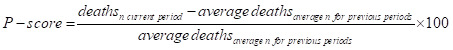



Subsequently, we employed a Bayesian approach to examine excess mortality during COVID-19 periods. We performed a log-linear model within a Bayesian framework using observed data between 2001–23, assuming death counts followed a Poisson distribution, to estimate the incidence rate ratio (IRR) for the peak (2020–21) and end-of-COVID-19 (2022–23) periods compared to the pre-COVID-19 period (2001–19). To estimate the IRR, we considered a variable with three categories in the model – pre-COVID-19, peak-COVID-19 and end-of-COVID-19 – using data between 2001–23.

We assessed the excess mortality as the difference between observed and predicted deaths based on the time series trends. We constructed a model using data on the monthly death counts before the onset of the COVID-19 pandemic. Then, we used this model to predict the expected death counts during and following the COVID-19 period under the assumption that the pandemic did not occur (counterfactual scenario). We then assessed the disparity between the predicted and actual death counts during the peak- and end of COVID-19 to measure the pandemic’s impact. After excess mortality estimation, we predicted future trends for the period 2024–30 based on the observed data between 2001–23. This prediction allowed us to assess the potential long-term impact of the pandemic on mortality patterns in the urban Bangladeshi community.

To avoid fitting problems in age groups with small death counts, we applied a Bayesian model [16], which also allowed for producing predictive and credible intervals. A log-linear model was used to fit the model and to predict the future mortality status, assuming the number of deaths for the ‘i’ age group at the ‘t’ time point following a Poisson distribution, as suggested by previous studies [16]. We employed Gaussian distributions as prior distributions for intercept and time covariate. The linear model was fitted initially, with monthly death count as the outcome variable and time as the covariate. We used the coefficient estimates and corresponding standard deviation (SD) as the mean (x̄) and SD of the prior distribution. As our data are a time series, we assessed the autocorrelation using the autocorrelation function with its lagged values. If any indication of autocorrelation was found, we employed autoregressive models to address this issue. Similarly, we adjusted seasonality in the monthly data set by incorporating seasonal patterns into the model to ensure accurate capture of periodic fluctuations (Appendix S1 in the **Online Supplementary Document**).

We reported the IRR with 95% credible interval (CrI) during peak- and end-of-COVID-19 periods. We also documented the x̄ and 95% CrI of predicted deaths for each month, both for excess mortality estimation in 2020–23 and for future prediction in 2024–30. Additionally, we reported yearly predicted values for the period 2024–30 by fitting a model with yearly death count as the outcome variable and time as the covariate. We considered two scenarios for our future predictions. Scenario one incorporates observed data between 2001–23, including the effects of the COVID-19 pandemic, while scenario two excludes the pandemic’s impact, using data between 2001–19. We provided these estimates for all age groups, including total population, neonates (zero to 28 days), children (29 days to five years), adolescents (six to 19 years), young adults (20–40 years), and middle to old age (≥40). We implemented the models using ‘inla’ package in *R*, version 4.3.3 (R Core Team, Vienna, Austria).

## RESULTS

The total number of deaths included in this analysis was 70 585. The largest proportion of deaths was recorded from Mirpur Buddhijibi, which accounted for 50% of the sample, followed by Rayer Bazar, with 21% of the deaths. Uttara Sector 14, established in 2019, had a smaller share, with 0.5% of the deaths. The majority of deaths were among adults aged >40 years, accounting for 65% of the total. Neonates comprised 14%, while young adults aged 20–40 years accounted for 9%. Adolescents aged six to 19 years comprised the smallest group, with 3% of the deaths (**Table 1**).

**Table 1:** Death distribution of the selected sample by graveyard and age*

Graveyard	Neonate (0-28 d)	Child (29 d to 5 y)	Adolescent (6–19 y)	Young adult (20–40 y)	Older adult (>40 y)	Total
Uttara Sector 12	267 (11.1)	106 (4.4)	54 (2.2)	151 (6.3)	1835 (76.1)	2413 (100)
Uttara Sector 14	35 (10.1)	8 (2.3)	9 (2.6)	26 (7.5)	269 (77.5)	347 (100)
Uttara Sector 4	517 (13.0)	286 (7.2)	130 (3.3)	274 (6.7)	2775 (69.7)	3982 (100)
Banani Graveyard	1832 (13.1)	1280 (9.2)	321 (2.3)	836 (6.0)	9705 (69.5)	13 974 (100)
Mirpur Buddhijibi	4558 (12.9)	3336 (9.5)	1362 (3.9)	3380 (9.6)	22 634 (64.2)	35 270 (100)
Rayer Bazar	2912 (20.0)	889 (6.1)	474 (3.3)	1448 (9.9)	8876 (60.8)	14 599 (100)
Total	10 121 (14.3)	5905 (8.4)	2350 (3.3)	6115 (8.7)	46 094 (65.3)	70 585 (100)

*Presented as n (%).

**Figure 1:**
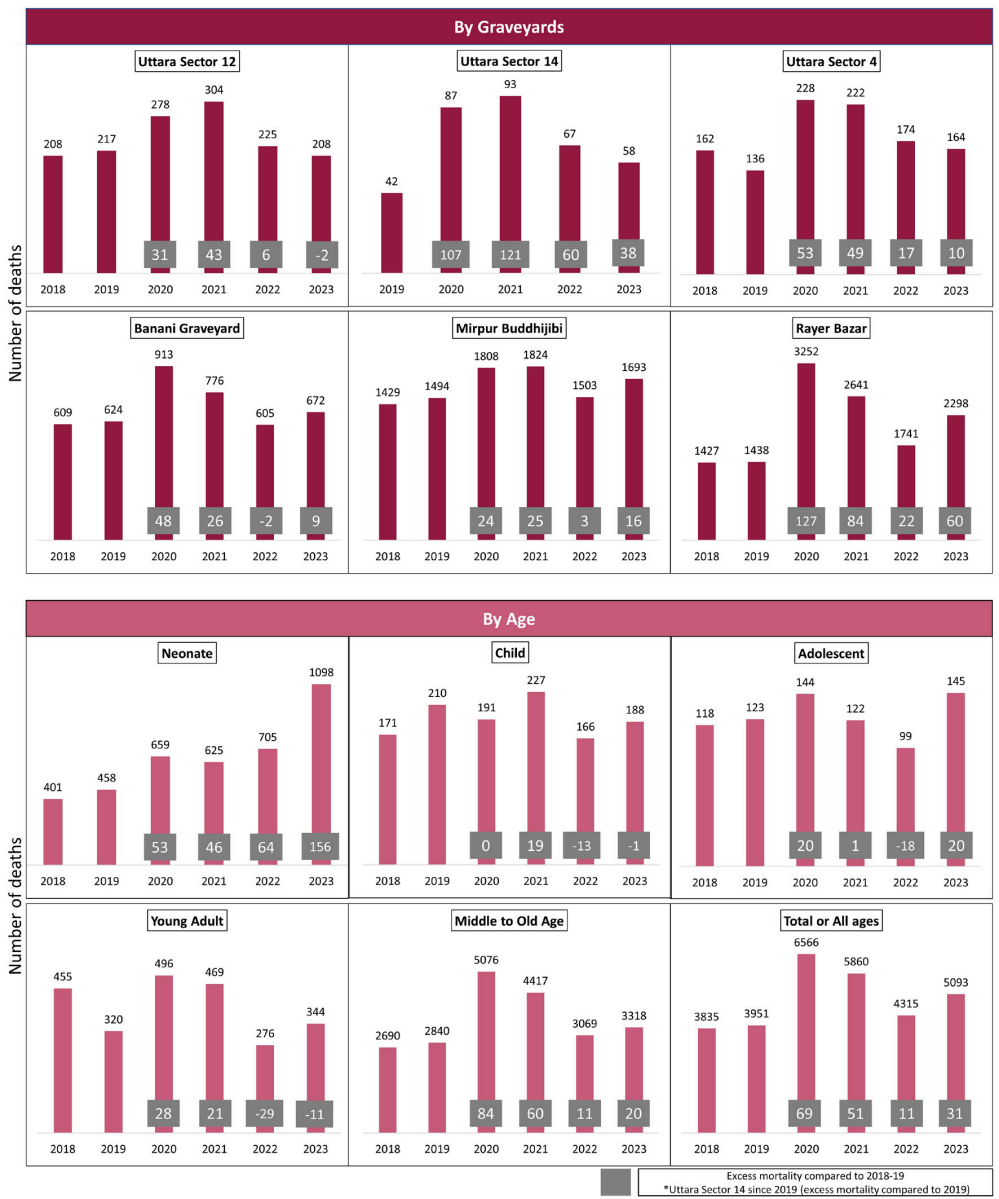
Yearly number of deaths by graveyards and age. Grey square with P-score values for assessing excess mortality between 2020–23 compared to the average of 2018 and 2019.

P-scores underscore the pandemic’s significant impact on death rates, especially in 2020–21 (**Figure 1**). While there was some decrease in subsequent years, all graveyards continued to experience excess deaths even after the pandemic (2022–23) except Uttara Sector 12. Mortality was 69% greater in 2020 and 31% in 2023 compared to the 2018–19 average. In Uttara Sector 12, the highest excess mortality was 43% in 2021 but declined to 2% lower in 2023 than in 2018–19. Banani Graveyard peaked at 48% excess mortality in 2020, decreasing to 9% in 2023. The highest excess mortality was in Rayer Bazar, with a P-score of 127% in 2020, decreasing to 60% in 2023. Neonates experienced a 53% increase in excess deaths in 2020, escalating to 156% in 2023 compared to the 2018–19 average. In the middle to old age group, excess deaths rose by 84% in 2020, declining to 20% in 2023.

The overall IRR for deaths was 1.66 times higher during the COVID-19 (95% CrI = 1.35–2.04) and 1.23 times higher in the end-of-COVID-19 period (95% CrI = 0.92–1.64) (**Table 2**). For neonates, IRR was significantly higher during (IRR = 1.45; 95% CrI = 1.02–2.05) and end-of-COVID-19 (IRR = 1.67; 95% CrI = 1.02–2.71) periods. We observed higher IRR during COVID-19 among young adults aged 20–40 years (IRR = 1.50; 95% CrI = 1.04–2.16) and individuals aged >40 years (IRR = 1.79; 95% CrI = 1.46–2.18).

**Table 2:** IRR with 95% CrI by age group estimates for the peak (2020–21) and end-of-COVID-19 (2022–23) compared to pre-COVID-19 (2001–19)

Age	Peak COVID 19, IRR (95% CrI)	End of COVID-19, IRR (95% CrI)
All ages	1.66 (1.35–2.04)	1.23 (0.92–1.64)
Neonate (0–28days)	1.45 (1.02–2.05)	1.67 (1.02–2.71)
Child (29 days to 5 years)	0.94 (0.61–1.45)	0.71 (0.39–1.30)
Adolescent (6–19 years)	1.15 (0.85–1.56)	1.01 (0.66–1.52)
Young adult (20–40 years)	1.50 (1.04–2.16)	0.90 (0.54–1.51)
Middle to old age (>40 years)	1.79 (1.46–2.18)	1.23 (0.93–1.65)

CrI – credible interval, IRR – incidence rate ratio

**Figure 2:**
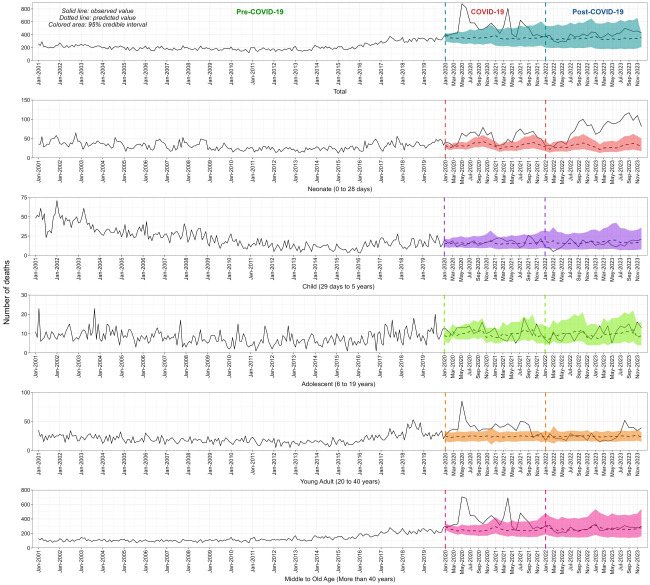
The monthly number of observed and predicted deaths (total, neonate, child, adolescent, young adult, middle to old age) for assessing excess mortality.

During the COVID-19 period, there was a notable increase in the number of observed deaths, surpassing the upper bounds of the predicted 95% CrI (**Figure 2**; Figure S3 in the **Online Supplementary Document****)**. Particularly in May 2020, there were 881 observed deaths compared to the predicted 355 (95% CrI = 258–439), followed by June 2020 with 477 excess deaths. Another peak occurred in April 2021, with 475 excess deaths. In the end-of-COVID-19 phase, observed deaths generally decreased compared to the peak during COVID-19.

Among neonates during the COVID-19 pandemic, there was a significant rise in deaths, exceeding the predicted range, with the highest excess deaths observed in July 2021 (n observed = 75, n predicted = 32; 95% CrI = 20–47). Excess mortality remained elevated in the end-of-COVID-19 phase, peaking in September 2023 (n observed = 117, n predicted = 34; 95% CrI = 20–56). For both children and adolescents, observed deaths fluctuated but mostly stayed within the predicted range across the phases. Among young adults, mortality rose during COVID-19, surpassing predicted credible intervals. This trend is less pronounced end-of-pandemic. In the middle to old age group, there was a substantial increase in observed deaths during the COVID-19 period, significantly exceeding the predicted range, with the highest excess deaths observed in May 2020 (n observed = 705, n predicted = 253; 95% CrI = 194–324) and then April 2021 (n observed = 690, n predicted = 236; 95% CrI = 156–347).

**Figure 3:**
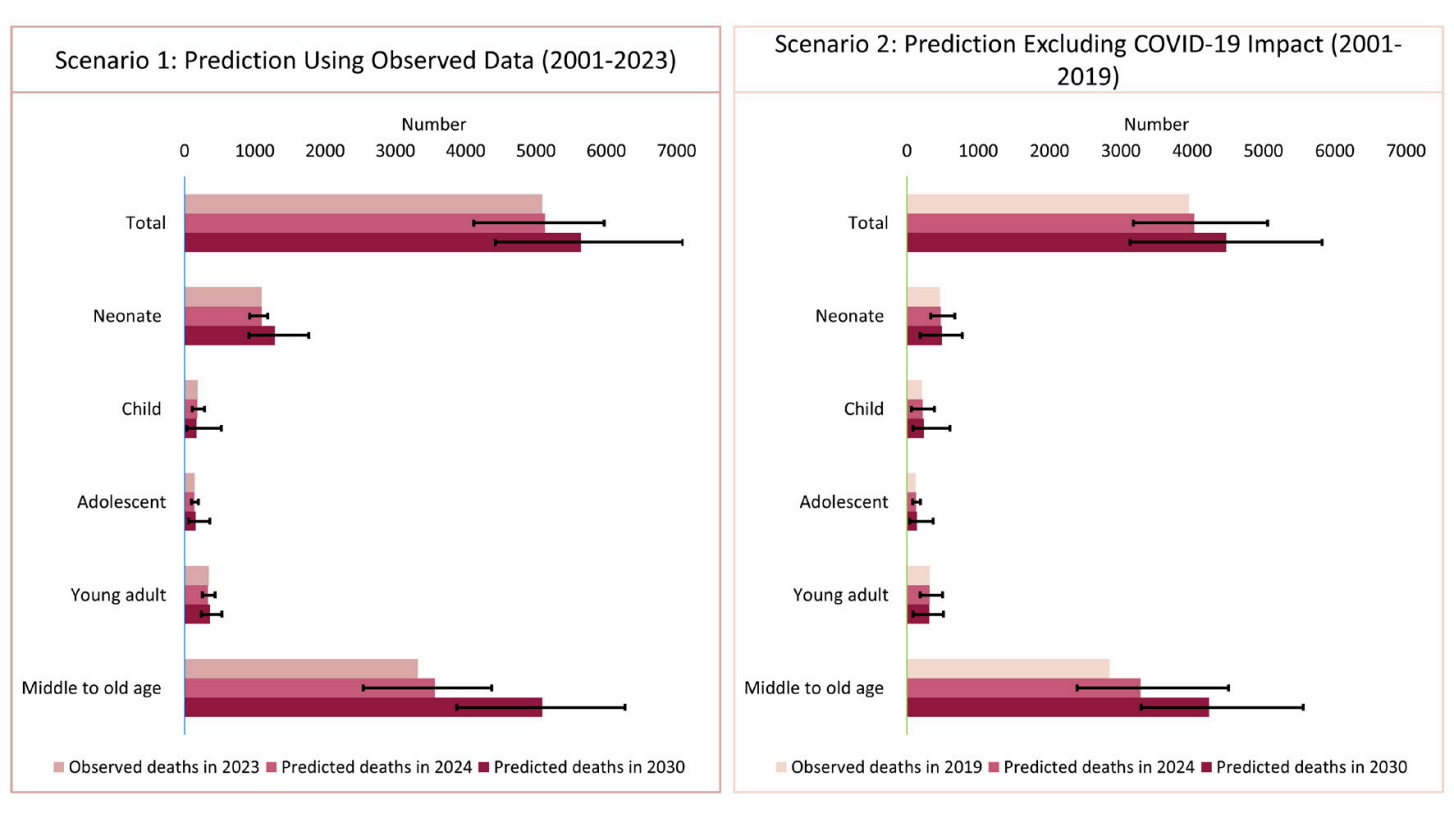
Yearly number of predicted deaths in 2024 and 2030, under two scenarios; scenario one for prediction using observed data (2001–23) and scenario two for prediction excluding COVID-19 impact (2001–19).

Under scenario one (considering observed data between 2001–23), the predicted deaths for 2024 are higher than the observed deaths in 2023 in several age groups, such as neonates (n observed = 1098, n predicted = 1101; 95% CrI = 922–1180) and the middle to old age group (n observed = 3318, n predicted = 3564; 95% CrI = 2540–4365), indicating an upward trend in mortality (**Figure 3**). This trend is projected to continue into 2030, with substantial increases among neonates (n predicted in 2030 = 1288; 95% CrI = 914–1769) and individuals aged >40 years (n predicted in 2030 = 5089 (95% CrI = 3871–6267). Scenario two, which excludes COVID-19 impact (considering observed data between 2001–19), also predicts a rise in deaths, although less pronounced compared to scenario one.

## DISCUSSION

Assessing the true impact of the COVID-19 pandemic on mortality, even post-pandemic, is critical for developing evidence-based health policies and enhancing preparedness for future pandemics. Our study, one of its first kinds in examining excess mortality caused by COVID-19 during the peak and end of pandemic periods (2020–23), uses graveyard-based death registration data from Dhaka, the capital city of Bangladesh. We addressed the gaps in future mortality predictions using graveyard records, while previous evidence outlined excess deaths that occurred earlier or during the pandemic periods [11–13]. We found an increase in deaths during peak-COVID-19 and also end-of-COVID-19 periods compared to deaths that happened in pre-COVID-19. According to our findings, during the pandemic period (2020–21), there was a notable increase in overall mortality, with IRR indicating deaths were 1.66 times higher compared to the pre-COVID-19 period (2001–19). This trend persisted into the end-of-COVID-19 period (2022–23), though at a slightly reduced rate (IRR = 1.23). We also observed that the pandemic’s effects varied across different age groups, with neonates showing increased mortality even in the end-of-pandemic period. Future predictions indicate increased mortality through 2030 among neonates and middle to older-aged people. This study established an efficient use of the graveyard records in explaining and predicting mortality for a specific locality.

Our findings align with previous studies conducted in Bangladesh and other developing countries, which have documented increased mortality rates during the COVID-19 pandemic [3,11,12,17–19]. One study conducted in Jamalpur town, northwest of Bangladesh using urban graveyard-based data, found that the average monthly death toll in an urban Bangladeshi area increased from 69 in the pre-COVID-19 period (January 2015–February 2020) to 92 during the COVID-19 period (March 2020–December 2021) [12]. Another study in rural area of Bangladesh noted elevated mortality rates among adults during the pandemic period in 2020 [11]. In addition, a study by Hossain et al. reported higher excess mortality through a household survey in rural areas [20]. Similarly, research from China observed increased mortality rates in Wuhan city during the COVID-19 period relative to the pre-COVID-19 era [21]. The significant increase in mortality during the COVID-19 pandemic can be attributed to multiple factors, including the direct effects of the virus, gaps in health care systems, and disruptions in routine health care services. These findings are consistent with global reports of increased mortality due to COVID-19 [3]. A study conducted in 11 sub-Saharan African countries observed that the COVID-19 pandemic limited service utilisation for 18% of study participants due to travel restrictions, health facility closures, and fear of infection [22]. Urban areas like Dhaka experienced higher excess mortality, potentially due to greater population density, poor hygiene measures and challenges in health care delivery with heightened strain on medical resources [23]. A study conducted in 2020 documented that, Dhaka, home to around 20 million people, was the country's epicentre of COVID-19 infections which can be a critical driver for the significant impact on health care services [23]. The implementation of strict lockdown measures, while necessary to curb the spread of the virus, also inadvertently hampered access to essential health care services. This is particularly evident in the observed delays and disruptions in routine medical care, which likely contributed to the elevated mortality rates. Moreover, the high population density and congested living conditions in urban slums made it difficult to implement effective social distancing and hygiene measures, exacerbating the spread of the virus and its associated mortality.

The highest excess deaths in our study from Dhaka North City Corporation graveyard records were observed in May 2020, followed by June 2020, coinciding with the first lockdown, with another peak in April 2021 during the second lockdown in Bangladesh. A similar study using graveyard-based data documented the highest deaths in August 2020 and then July 2021 [12], similar to our findings. WHO estimated that excess mortality in Bangladesh, initially surged during the June to August period of 2020, over 30 000 additional deaths beyond the expected numbers under normal conditions. In the second year, the months of April, and June to August experienced the highest mortality rates. April 2021 is when the highly transmissible Delta variant of the deadly virus is suspected to have entered Bangladesh [24]. By the end of May 2021, the Delta variant constituted 68% of the variants circulating in Dhaka city [25]. The monthly excess deaths estimated in this study show a strong correlation with COVID-19 fatalities, rising and falling with the waves of the epidemic. This is not to say that all the excess deaths were due to COVID-19; there may have been some deaths from other causes as well. These figures include deaths caused either directly by the COVID-19 or fatalities resulting from the pandemic's impact on health care systems, such as cancer patients who couldn't receive treatment when hospitals were overwhelmed with COVID-19 cases. However, comparing the excess deaths estimates due to COVID-19 mortality based on disease spread with the expected numbers under normal circumstances suggests that the majority of these excess deaths were likely from COVID-19 [26].

Our study also showed an increasing trend in mortality beyond the COVID-19 period, its magnitude was lower compared to the peak of COVID period. The continued excess mortality in the end-of-pandemic period suggests long-term health consequences, possibly due to delayed medical treatments, other emerging infections, economic hardships, and ongoing health system challenges exacerbated by the pandemic. For instance, a household survey conducted in Bangladesh revealed that individuals in the lowest wealth category had a mortality rate that was significantly twice as high compared to those in higher wealth categories [20]. Countries like Turkey and Tunisia experienced increased mortality in the end-of-pandemic period (2022–23), as indicated by P-scores derived from data in the Human Mortality Database (2024) and the World Mortality Data set (2024) [27]. The growing burden of multimorbidity, and limited access to affordable health care, the economic repercussions of the pandemic are likely to have long-term impacts on health, which are not fully captured in short-term estimates of excess mortality [28,^29^].

The persistent excess mortality observed among older adults and neonates in this study is particularly concerning. The Bangladesh Sample Vital Statistics 2022 also documented that the age-specific death rate was higher among infants under one year and elderly individuals compared to other age groups [30]. COVID-19 infection tends to affect the elderly more severely than younger individuals [31,32], and pre-existing medical conditions such as cardiovascular disease, diabetes, high blood pressure, and respiratory diseases might lead to death due to COVID-19. In low-income, developing, and densely populated countries like Bangladesh, the aging population are especially vulnerable due to insufficient health services, socio-economic challenges, environmental settings, cultural practices, personal hygiene habits, and a lack of proactive approaches to preventing infectious diseases [33]. Bangladesh’s older adults are generally considered vulnerable because of their dependence on family, limited ability to access health services, multiple health conditions, and inadequate social security programmes [34,35].

Neonates, being extremely vulnerable, likely suffered from disruptions in maternal and newborn health care services during the pandemic. Evidence from a study based on District Health Information Software, version two, reported a significant decline in maternal health service utilisation during the pandemic, with approximately 30% reductions in antenatal care visits, institutional deliveries, and caesarean sections compared to the pre-pandemic period (2017–19) [36]. Another study found a notable decline in vaccine administration – including Bacille Calmette-Guérin, pentavalent third dose, and measles vaccinations – in April 2020 compared to the pre-pandemic period of 2017–19 [37]. Furthermore, vaccination programs also faced significant challenges during the pandemic. A notable decline in measles vaccine administration was observed in January 2021, exacerbated by a nationwide health worker strike beginning in late 2020 [38]. The protests by health workers demanding a pay increase halted routine immunisation across the country, impacting millions of lives. This strike disrupted routine immunisation programs, increasing the risk of vaccine-preventable diseases among neonates and young children. Delays in receiving critical care can significantly increase neonatal mortality. The strain on health care resources during the pandemic may have led to delayed or foregone treatments for non-COVID-19 illnesses, contributing to excess mortality in this age group. Many patients preferred to stay at home, fearing maltreatment in hospitals [39], with a report indicating that 79% of patients in Bangladesh received treatment over the phone [40]. Maternal and neonatal mortality rates in government hospitals significantly increased during the two years of the COVID-19 outbreak, with neonatal death rates rising by 1% in 2020 and 6% in 2021 [41]. Various factors contributed to the increase in deaths, such as critical health conditions caused by inadequate care prior to delivery, lack of medical care at hospitals during childbirth, giving birth in unhygienic environments, infants born with low birth weight, and insufficient neonatal care. During these years, the health care system was heavily focused on COVID-19, with less attention given to maternal and neonatal care, resulting in many mothers not receiving adequate pre-birth and post-birth care [41].

Our study, using data between 2001–23, predicts mortality trends in Dhaka up to 2030, including a scenario with COVID-19 (2001–23) and one without COVID-19 (2001–19). We observed an increasing mortality trend, with deaths rising from 2014 to 2019 after a decline between 2001–13. National estimates from the Sample Vital Registration System show a decreasing crude death rate (CDR) from 2001–19, but an increase from 2020–22, with neonatal mortality rate decreasing yet rising again in 2021–22. In Dhaka, the CDR per 1000 population began to rise pre-pandemic (2018 CDR = 2.0, 2019 CDR = 3.3, 2020 CDR = 3.9, 2021 CDR = 3.6, 2022 CDR = 4.0), aligning with our findings [30,42–46].

However, we acknowledge graveyard data may not fully represent the population, as many residents are buried in their native villages. Despite these limitations, our findings highlight critical mortality concerns, particularly among neonates and older adults. The increasing CDR in Dhaka underscores the need to address health system weaknesses. Strengthening health care infrastructure, ensuring continuous access to essential services, and enhancing public health strategies are crucial. Targeted interventions for vulnerable populations are necessary to mitigate the pandemic's long-term impacts and achieve Sustainable Development Goals.

### Implications for policy and practice

The study underscores the value of graveyard-based death registration data for monitoring mortality trends and overall health status, particularly during crises like the COVID-19 pandemic. This data provides timely and reliable mortality patterns, which can enhance national health monitoring systems' accuracy and offer critical insights into public health impacts. Policymakers and public health officials can leverage this data to understand the direct and indirect effects of crises, aiding in evidence-based decision-making. Institutionalising this data collection can improve health surveillance and crisis response. However, limitations include potential underreporting and challenges in accurately attributing causes of death. Sustained use of this data can strengthen health systems, improve public health outcomes, and enhance preparedness for future pandemics, contributing to achieving Sustainable Development Goals related to health and well-being. The study also highlights the need to strengthen health systems for resilient and responsive health care delivery during crises, especially in urban areas like Dhaka. Expanding the capacity and accessibility of primary health care facilities is essential to reduce the burden on tertiary hospitals. Additionally, establishing improved emergency response systems, including rapid ambulance services and emergency care units, can help address health crises more effectively. Integrating digital health platforms, such as telemedicine, can ensure continued access to care during disruptions like pandemics. Targeted interventions for vulnerable populations, including slum dwellers and low-income groups, are necessary to provide equitable health care services through subsidised programmes and focused health campaigns. The observed excess mortality and predicted trends up to 2030 indicate ongoing challenges, particularly among vulnerable populations such as neonates and older adults. For older adults, strengthening geriatric health care services and addressing age-related comorbidities are essential to mitigate the observed trends. For neonates, ensuring uninterrupted maternal and newborn care services, improving neonatal care units, and expanding health care accessibility are crucial policy priorities. Targeted health policies, robust health infrastructure, continuous access to essential services, and enhanced public health communication are crucial to mitigating future impacts and achieving long-term health improvements.

### Strengths and limitations

This study presents several strengths, including a rigorous analysis of urban mortality trends and the novel use of graveyard-based death registration data to estimate excess mortality during peak- and end-of-COVID-19 periods in Bangladesh. This approach is particularly valuable when conventional health reporting systems are disrupted. Graveyard-based data, although often underutilised, can provide important insights into mortality trends, especially in settings where other mortality surveillance systems may be incomplete or unavailable. While we recognize that national death registries or hospital-based data are typically the gold standard for mortality tracking, graveyard-based data can serve as a valuable complement in areas where these systems are lacking or where access to health care is limited. In the context of the COVID-19 pandemic, graveyard-based data offers a unique opportunity to estimate excess mortality in populations where health service disruptions may affect the reporting of deaths. Methodologically, the study employs robust statistical techniques, enhancing the reliability of its findings compared to traditional time-series models. We recognise that the P-score, as a metric, is dependent on historical baselines and may not account for pre-existing mortality trends or structural changes in urban demographics and health care. However, the Bayesian approach was implemented to address this issue. By comparing the observed and predicted mortality under different scenarios (with and without the impact of COVID-19), we provide a more nuanced analysis that goes beyond the limitations of the P-score alone. Gaussian priors were employed for intercepts and covariates, and we checked the goodness of fit of the model using the deviance information criterion to assess the better choice of the priors. Autocorrelation and seasonality in the time-series data were addressed using autoregressive models and adjustments for seasonal patterns, ensuring that these factors did not bias our results. The study’s detailed age-specific analyses identify vulnerable populations and areas needing targeted interventions, making the findings highly relevant for policy and practice. By examining mortality trends over several years, the study provides a longitudinal perspective that captures both the immediate and long-term impacts of the pandemic, offering valuable insights for future public health strategies and resource allocation. These strengths contribute to a comprehensive understanding of mortality dynamics and inform future policy formulation and public health research in Bangladesh.

We acknowledge several limitations in our study. It primarily reflects urban mortality trends in the public graveyards of Dhaka North City Corporation, which may not be generalisable to other parts of Dhaka, other rural areas or the national context. However, urban areas like Dhaka often serve as public health epicentres, offering valuable early indicators of broader trends. Dhaka North City Corporation covers a substantial proportion of the population of this capital city. These findings may provide insights into urban mortality patterns that could be applicable to other metropolitan areas in Bangladesh with similar health care infrastructure and sociodemographic profiles. To enhance generalisability in future research, it will be essential to integrate data from other urban and rural graveyards. Such efforts could help identify urban-rural disparities and provide a more comprehensive understanding of mortality trends across Bangladesh. The reliability of our graveyard-based data in Dhaka city can vary due to inconsistent death registrations, inadequate record-keeping, potential underreporting and the exclusion of individuals buried outside Dhaka. The lack of detailed cause-of-death information limits distinguishing between COVID-19-related and other deaths. However, our primary objective was to estimate excess mortality during COVID-19, which is an overall impact of the pandemic. To address the potential influence of COVID-19, we implemented scenario-based analyses to provide a counterfactual perspective, estimating what mortality trends would have looked like without the impact of COVID-19 (based on pre-pandemic data between 2001–19). The first scenario assumes that the trends observed during the COVID-19 period, including excess mortality, will continue in the future, while the second scenario assumes a return to pre-pandemic conditions. This dual approach strengthens the robustness of our findings and minimises reliance on cause-specific data. Additionally, the accuracy of other covariates may be compromised by reporting errors or incomplete information, restricting comprehensive mortality analysis. For instance, while gender disparities in mortality and health care access are widely recognised, our study faced limitations due to incomplete or missing gender-specific mortality data. This constraint limited our ability to explore gender-based trends in detail. However, this finding underscores the need to improve the quality and completeness of gender-specific mortality data in future surveillance systems to better understand and address gender disparities during health crises. In addition, the graveyard register records we used is limited to variables such as sex, religion, age, reported cause of death, and place of death, making it challenging to directly analyse the disparities due to socio-economic status. Biases in data collection practices, including varying recording standards across regions and cemeteries, may lead to uneven mortality trend representation. Integrating multiple data sources is recommended for a more robust understanding of mortality patterns. While our statistical models are robust, inherent data uncertainties may impact the precision of mortality predictions. Therefore, caution is advised when interpreting these results, and further large-scale studies are recommended to enhance the reliability of graveyard-based data for mortality research. In this study, we aimed to provide policymakers with crucial insights for strategic planning and to offer researchers the potential of this valuable data for future studies rather than delivering perfect predictions and precise impacts.

## CONCLUSIONS

In this study, we critically examined excess mortality during and after the COVID-19 pandemic using graveyard-based death records from Dhaka, Bangladesh. The findings reveal an indication of an increase in mortality during the pandemic, with elevated death rates persisting endemic, particularly among neonates and older adults. Predictions indicate a continued rise in mortality through 2030, highlighting the pandemic’s long-term health impacts. The study underscores the need for robust health infrastructure and continuous access to essential health services to mitigate these long-term effects. The innovative use of graveyard-based data offers important insights into urban mortality trends, demonstrating its potential for monitoring population health, especially during crises and in resource-limited settings. However, further studies with larger, more diverse samples are necessary to enhance the reliability and generalisability of the results.

## Additional material


Online Supplementary Document
