# Erratum: Akter E, Hossain AT, Ahamed B, Rahman MH, Hossain AKMT, Barua U, Islam MS, Manna RM, Hossain MA, Ara T, Usmani NG, Chandra P, Ameen S, Jabeen S, Ahmed A, Rahman TZ, Hassan MMU, Islam A, Barr BT, Rahman QS, Arifeen SE, Rahman AE. Excess mortality during COVID-19 and prediction of mortality in Bangladesh: an analysis based on death records in urban graveyards. J Glob Health. 2025;15:04050.

**DOI:** 10.7189/jogh.15.1504050err1

**Published:** 2025-03-07

**Authors:** Journal of Global Health Editorial Team



Following the publication of the manuscript ‘Excess mortality during COVID-19 and prediction of mortality in Bangladesh: an analysis based on death records in urban graveyards’ on 28 February 2025, the authors noted several errors in the main text and the reference list of the online version of the article which were absent from the PDF version, and vice versa. This discrepancy occurred during the final stage of production, where the authors’ corrections to the galley proof were not fully inputted within the article XML. It thus affected both the version displayed on the Journal’s website and PubMed Central. The editorial team regrets this error, takes full responsibility, and apologises for any inconvenience it may have caused to the readers or the authors.

To ensure consistency, these corrections, which we list below in detail, have been made to the online and the PDF version of the manuscript on 7 March 2025, following the authors’ notice on 3 March 2025.

## CORRECTIONS TO ONLINE VERSION ONLY

The following corrections have been made to align the online version of the article to the PDF.

Prior version, first paragraph of the ‘Discussion’ section: ‘According to our findings, during the pandemic period (202–21), there was a notable increase in overall mortality, with IRR indicating deaths were 1.66 times higher compared to the pre-COVID-19 period (2001–19).’

Revised version, first paragraph of the ‘Discussion’ section: ‘According to our findings, during the pandemic period (2020–21), there was a notable increase in overall mortality, with IRR indicating deaths were 1.66 times higher compared to the pre-COVID-19 period (2001–19).’

Prior version, seventh paragraph in the ‘Discussion’ section: ‘Maternal and neonatal mortality rates in government hospitals significantly increased during the two years of the COVID-19 outbreak, with neonatal death rates rising by 1% and 6% [41].’

Revised version, seventh paragraph in the ‘Discussion’ section: ‘Maternal and neonatal mortality rates in government hospitals significantly increased during the two years of the COVID-19 outbreak, with neonatal death rates rising by 1% in 2020 and 6% in 2021 [41].’

### References in prior version

4 Davidson JA, Hosie H. Limitations of pulse oximetry: respiratory insufficiency–a failure of detection. BMJ. 1993;307:372–3. Medline:8374423 doi:10.1136/bmj.307.6900.372

5 World Population Review. 2024. Available: https://worldpopulationreview.com/world-cities/dhaka-population. Accessed: 12 September 2024.

14 Bangladesh Bureau of Statistics. Report on the Survey of Private Healthcare Institutions 2019. Statistical Report. Dhaka, Bangladesh: BBS; 2021. Available: https://bbs.portal.gov.bd/sites/default/files/files/bbs.portal.gov.bd/page/b343a8b4_956b_45ca_872f_4cf9b2f1a6e0/2021-06-30-04-58-22ea330d54a12a1b26bb608d43130b91.pdf. Accessed: 14 September 2024.

15 Rahman AE, Hossain AT, Nair H, Chisti MJ, Dockrell D, El Arifeen S, et al. Prevalence of hypoxaemia in children with pneumonia in low-income and middle-income countries: a systematic review and meta-analysis. Lancet Glob Health. 2022;10:e348–59. Medline:35180418 doi:10.1016/S2214-109X(21)00586-6

27 Graham HR, Kamuntu Y, Miller J, Barrett A, Kunihira B, Engol S, et al. Hypoxaemia prevalence and management among children and adults presenting to primary care facilities in Uganda: a prospective cohort study. PLOS Glob Public Health. 2022;2:e0000352. Medline:36962209 doi:10.1371/journal.pgph.0000352

28 Mahmud I, Das S, Khan SH, Faruque A, Ahmed T. Gender disparity in care-seeking behaviours and treatment outcomes for dehydrating diarrhoea among under-5 children admitted to a diarrhoeal disease hospital in Bangladesh: an analysis of hospital-based surveillance data. BMJ Open. 2020;10:e038730. Medline:32883737 doi:10.1136/bmjopen-2020-038730

30 Fuchs A, Bielicki J, Mathur S, Sharland M, Van Den Anker JN. Reviewing the WHO guidelines for antibiotic use for sepsis in neonates and children. Paediatr Int Child Health. 2018;38:S3–15. Medline:29790842 doi:10.1080/20469047.2017.1408738

40 El Arifeen S, Hossain AT, Rahman AE. Detecting hypoxaemia among children with pneumonia in low-resource settings. Lancet Respir Med. 2023;11:756–7. Medline:37657850 doi:10.1016/S2213-2600(23)00300-4

41 Chisti MJ, Salam MA, Smith JH, Ahmed T, Pietroni MA, Shahunja K, et al. Bubble continuous positive airway pressure for children with severe pneumonia and hypoxaemia in Bangladesh: an open, randomised controlled trial. Lancet. 2015;386:1057–65. Medline:26296950 doi:10.1016/S0140-6736(15)60249-5

42 Arifeen SE, Bryce J, Gouws E, Baqui A, Black R, Hoque D, et al. Quality of care for under-fives in first-level health facilities in one district of Bangladesh. Bull World Health Organ. 2005;83:260–7. Medline:15868016

43 Anwar I, Kalim N, Koblinsky M. Quality of obstetric care in public-sector facilities and constraints to implementing emergency obstetric care services: evidence from high-and low-performing districts of Bangladesh. J Health Popul Nutr. 2009;27:139–55. Medline:19489412 doi:10.3329/jhpn.v27i2.3327

44 Chowdhury S, Hossain SA, Halim A. Assessment of quality of care in maternal and newborn health services available in public health care facilities in Bangladesh. Bangladesh Med Res Counc Bull. 2009;35:53–6. Medline:20120780 doi:10.3329/bmrcb.v35i2.3044

45 Hossain AT, Ameen S, Salim N, Ashish K, Ruysen H, Tahsina T, et al. Measuring coverage and quality of supportive care for inpatient neonatal infections: EN-BIRTH multi-country validation study. J Glob Health. 2022;12:04029. Medline:35486705 doi:10.7189/jogh.12.04029

46 Rahman AE, Hossain AT, Zaman SB, Salim N, KC A, Day LT, et al. Antibiotic use for inpatient newborn care with suspected infection: EN-BIRTH multi-country validation study. BMC Pregnancy Childbirth. 2021;21:229. Medline:33765948 doi:10.1186/s12884-020-03424-7

### References in revised version

4 COVID-19 Dynamic Dashboard for Bangladesh. 2024. Available: https://dashboard.dghs.gov.bd/pages/covid19.php. Accessed: 12 September 2024.

5 Dhaka Tribune. WHO estimates Covid-19 death toll in Bangladesh 5 times higher than official figures. 2024. Available: https://www.dhakatribune.com/bangladesh/health/269673/who-estimates-covid-19-death-toll-inbangladesh-5. Accessed: 12 September 2024.

14 Bangladesh Bureau of Statistics. Population and Housing Census 2022. Dhaka, Bangladesh: Bangladesh Bureau of Statistics; 2023. Available: https://bbs.portal.gov.bd/sites/default/files/files/bbs.portal.gov.bd/page/b343a8b4_956b_45ca_872f_4cf9b2f1a6e0/2024-01-31-15-51-53c55dd692233ae401ba013060b9cbb.pdf. Accessed:14 September 2024.

15 Dhaka Tribune. Dhaka ranks world’s sixth most populous city. 2024. Available: https://www.dhakatribune.com/bangladesh/261898/dhaka-ranks-world%E2%80%99s-sixth-most-populous-city. Accessed: 16 September 2024.

27 Mathieu E, Ritchie H, Rodés-Guirao L, Appel C, Gavrilov D, Giattino C, et al. Excess mortality during the Coronavirus pandemic (COVID-19). Our World in Data. 2020. Available: https://ourworldindata.org/excess-mortality-covid. Accessed: 22 September 2024.

28 World Health Organization, Regional Office for South-East Asia. 2019 Health SDG Profile: Bangladesh. 2021. Available: https://iris.who.int/handle/10665/327747. Accessed: 23 September 2024.

30 Bangladesh Bureau of Statistics. Bangladesh Sample Vital Statistics 2022. Dhaka, Bangladesh: Bangladesh Bureau of Statistics; 2023. Available: https://bbs.portal.gov.bd/sites/default/files/files/bbs.portal.gov.bd/page/b343a8b4_956b_45ca_872f_4cf9b2f1a6e0/2024-02-12-09-14-e96115a9e06fa25854d494fc9ffbde52.pdf. Accessed: 23 September 2024.

40 Morol S. 79% of patients get treatment over the phone. Daily Prothom Alo. 16 May 2020. Available: https://is.gd/cIhzS0. Accessed: 25 September 2024.

41 Akhter N. Maternal and neonatal deaths increase during two years of Covid. Daily Prothom Alo. 13 September 2022. Available: https://en.prothomalo.com/bangladesh/1g50fgl2yz. Accessed: 25 September 2024.

42 Bangladesh Bureau of Statistics. Bangladesh Sample Vital Statistics 2017. Dhaka, Bangladesh: Bangladesh Bureau of Statistics; 2018. Available: https://bbs.portal.gov.bd/sites/default/files/files/bbs.portal.gov.bd/page/6a40a397_6ef7_48a3_80b3_78b8d1223e3f/SVRS_2017.pdf. Accessed: 30 September 2024.

43 Bangladesh Bureau of Statistics. Bangladesh Sample Vital Statistics 2018. Dhaka, Bangladesh: Bangladesh Bureau of Statistics; 2019. Available: https://bbs.portal.gov.bd/sites/default/files/files/bbs.portal.gov.bd/page/6a40a397_6ef7_48a3_80b3_78b8d1223e3f/SVRS_Report_2018_29-05-2019%28Final%29.pdf. Accessed: 30 September 2024.

44 Bangladesh Bureau of Statistics. Bangladesh Sample Vital Statistics 2019. Dhaka, Bangladesh: Bangladesh Bureau of Statistics; 2019.

45 Bangladesh Bureau of Statistics. Bangladesh Sample Vital Statistics 2020. Dhaka, Bangladesh: Bangladesh Bureau of Statistics; 2021. Available: https://bbs.portal.gov.bd/sites/default/files/files/bbs.portal.gov.bd/page/6a40a397_6ef7_48a3_80b3_78b8d1223e3f/2021-06-30-04-37-90c4374ce2c14b93852ae7830f7ec3c1.pdf. Accessed: 30 September 2024.

46 Bangladesh Bureau of Statistics. Bangladesh Sample Vital Statistics 2021. Dhaka, Bangladesh: Bangladesh Bureau of Statistics; 2023. Available: https://drive.google.com/file/d/1HfirfErmcD6XEVDbgH0ubKe_TRyprrHM/view. Accessed: 30 September 2024.

## ONLINE AND PDF VERSION

The following corrections have been made to both the PDF and the online version of the article.

Prior version, footnote to Table 1: ‘*Presented as n (%) unless specified otherwise’

Revised version, footnote to Table 1: ‘*Presented as n (%).’

Prior version, legend to Figure 1: ‘Figure 1. Yearly number of predicted deaths in 2024 and 2030, under two scenarios; Scenario one for prediction using observed data (2001–23) and scenario two for prediction excluding COVID-19 impact (2001–19).’

Revised version, legend to Figure 1: ‘Figure 1. Yearly number of deaths by graveyards and age. Grey square with P-score values for assessing excess mortality between 2020–23 compared to the average of 2018 and 2019.’

Prior version, legend to Figure 3: ‘Figure 3. Yearly number of predicted deaths in 2024 and 2030, under two scenarios Scenario one for prediction using observed data (2001–23) and scenario two for prediction excluding COVID-19 impact (2001–19).’

Revised version, legend to Figure 3: ‘Figure 3. Yearly number of predicted deaths in 2024 and 2030, under two scenarios; scenario one for prediction using observed data (2001–23) and scenario two for prediction excluding COVID-19 impact (2001–19).’

