# Supplementary material for: Excess mortality during COVID-19 and prediction of mortality in Bangladesh: an analysis based on death records in urban graveyards
Source: J Glob Health. 2025 Feb 28;15:04050. doi: 10.7189/jogh.15.04050 (PMC11869516; doi:10.7189/jogh.15.04050)
Supplement: Online Supplementary Document [file jogh-15-04050-s001.pdf]

Supplement to: Akter E, Hossain AT, Ahamed B, Rahman MH, Hossain AKMT, Barua U, Islam MS, Manna RM, Hossain MA, Ara T, Usmani NG, Chandra P, Ameen S, Jabeen S, Ahmed A, Rahman TZ, Hassan MMU, Islam A, Barr BT, Rahman QS, Arifeen SE, Rahman AE. Excess mortality during COVID-19 and prediction of mortality in Bangladesh: an analysis based on death records in urban graveyards. J Glob Health. 2025;15:04050.

Figure S1: Death records extracted from the six graveyards, presented in number.

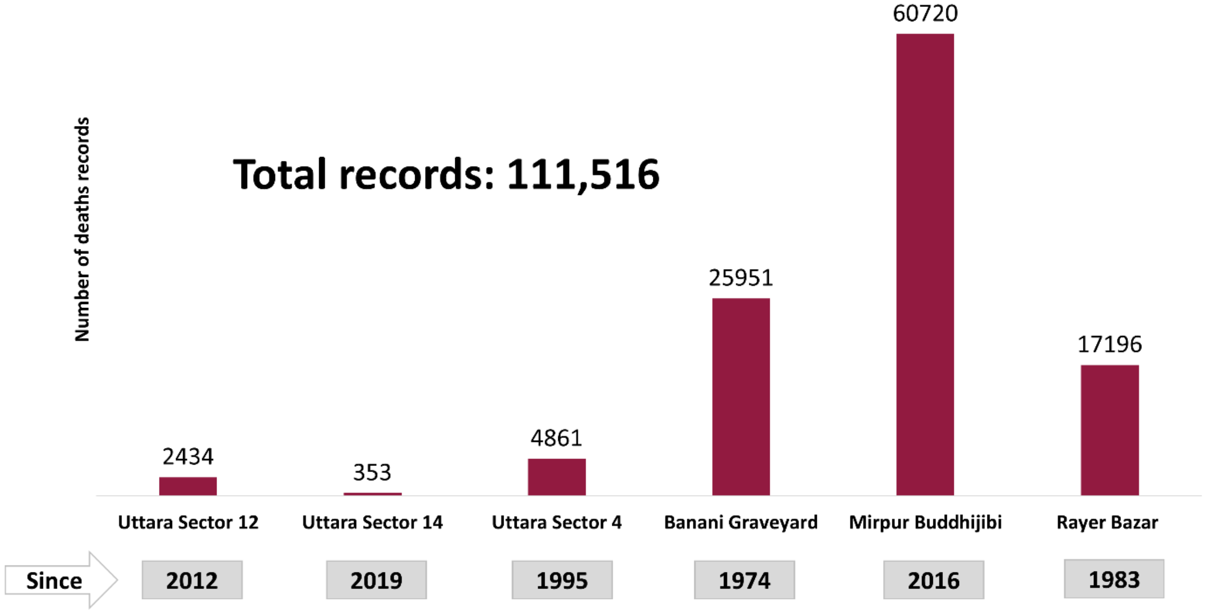

Figure S2: Sample selection process in the study.

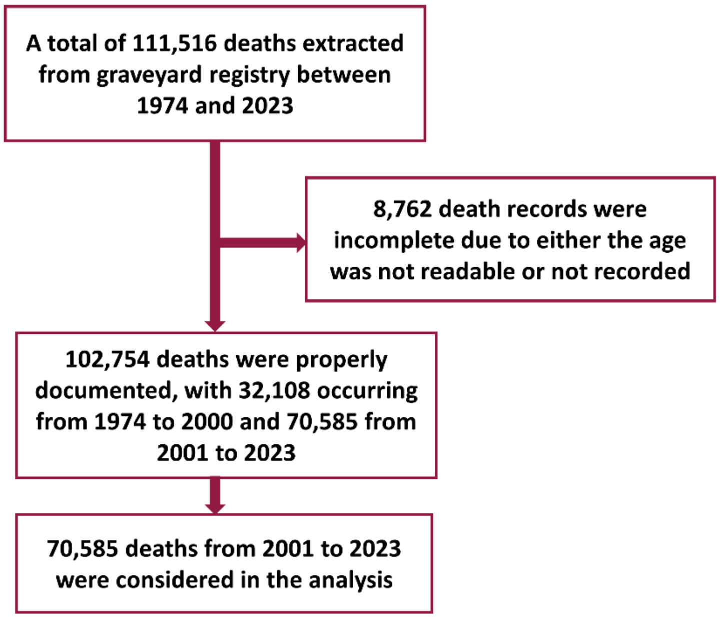

Figure S3: Differences in the monthly number of observed and predicted deaths (total, neonate, child, adolescent, young adult, middle to old age) for assessing excess mortality.

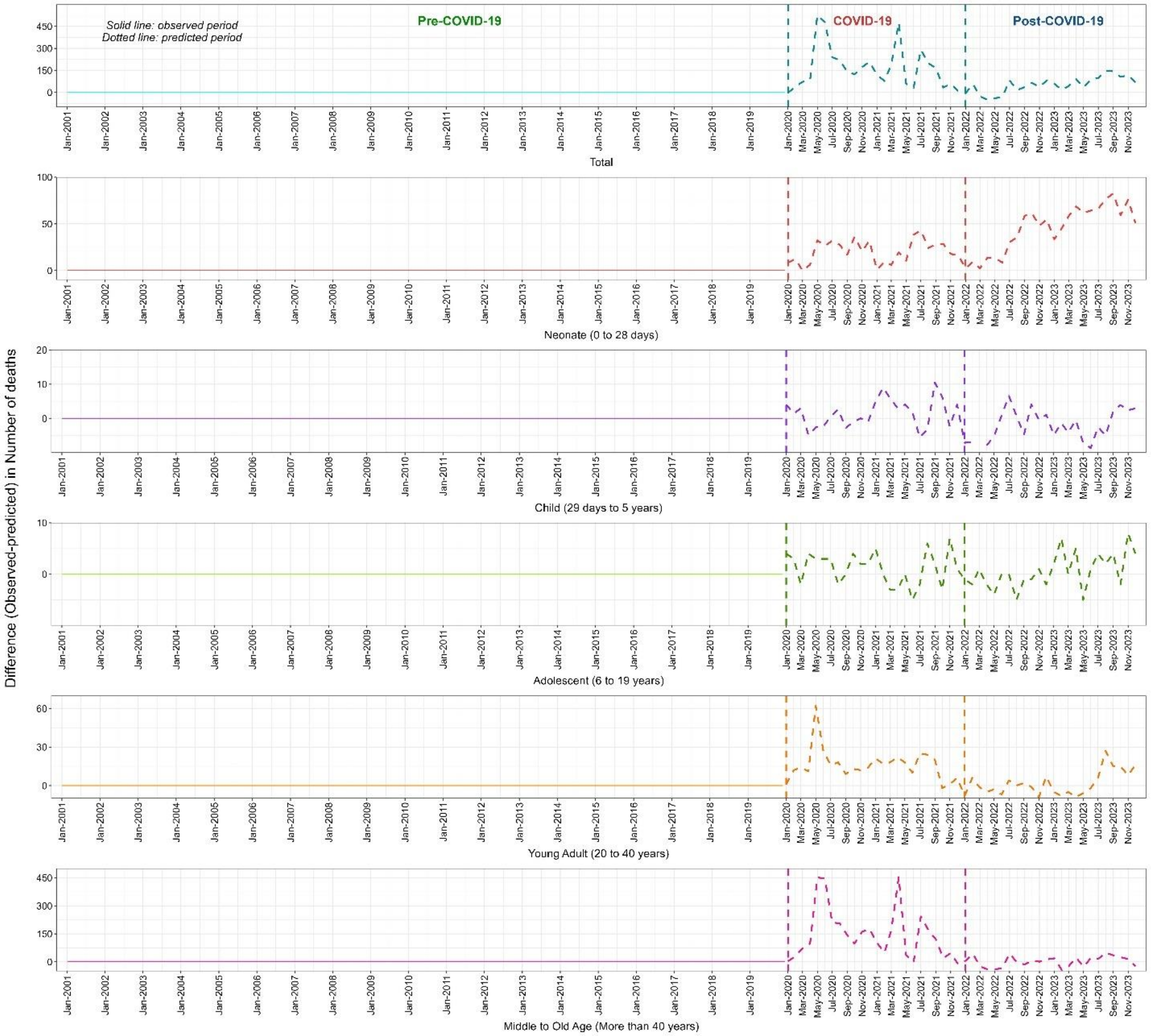

Figure S4: Monthly number of predicted deaths from 2024 to 2030, under scenario 1 (observed data from 2001 to 2023, including the COVID-19 pandemic's impact).

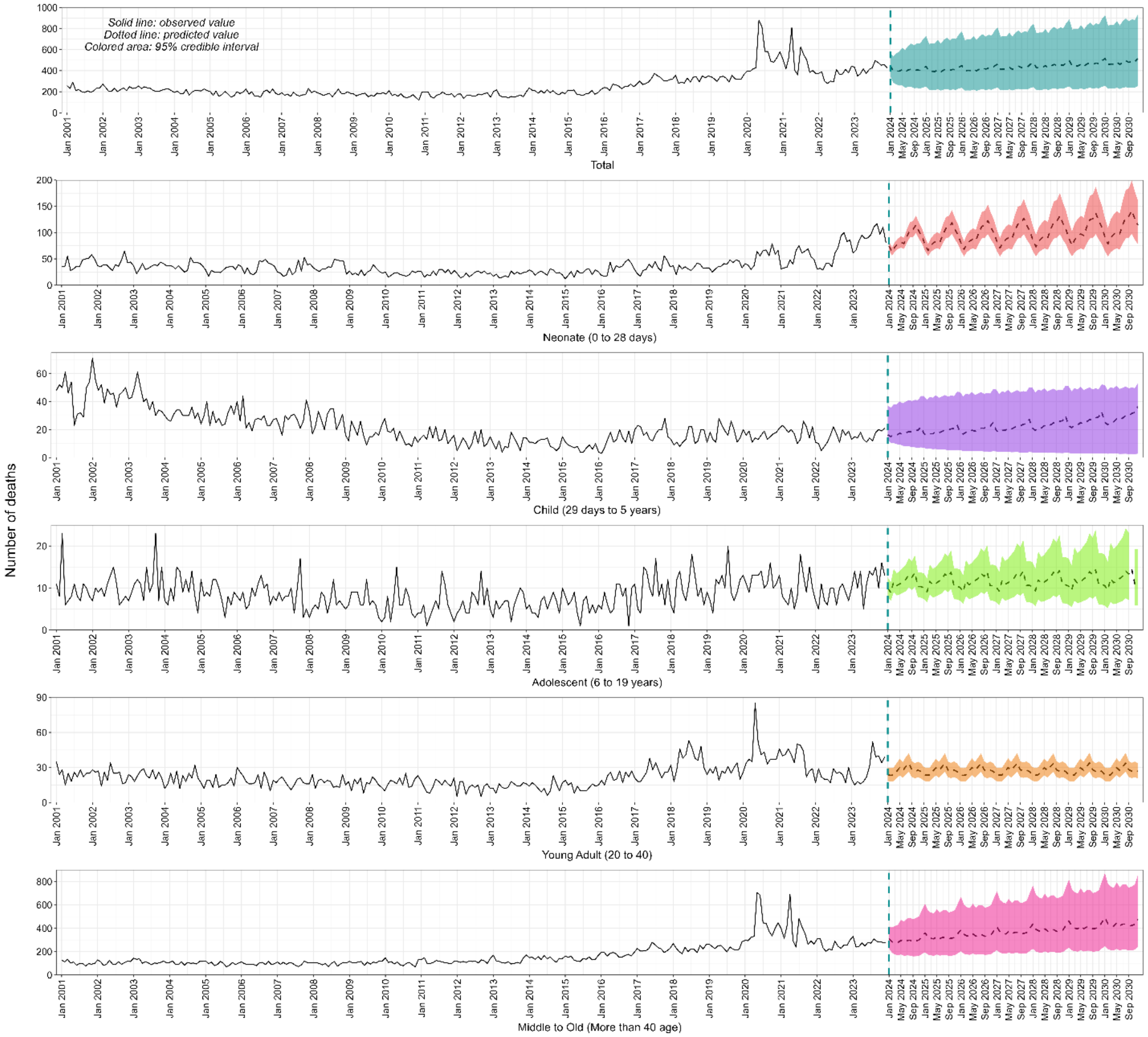

Figure S5: Monthly number of predicted from 2020 to 2030, under scenario 2 (observed data from 2001 to 2019, excluding the COVID-19 pandemic's impact).

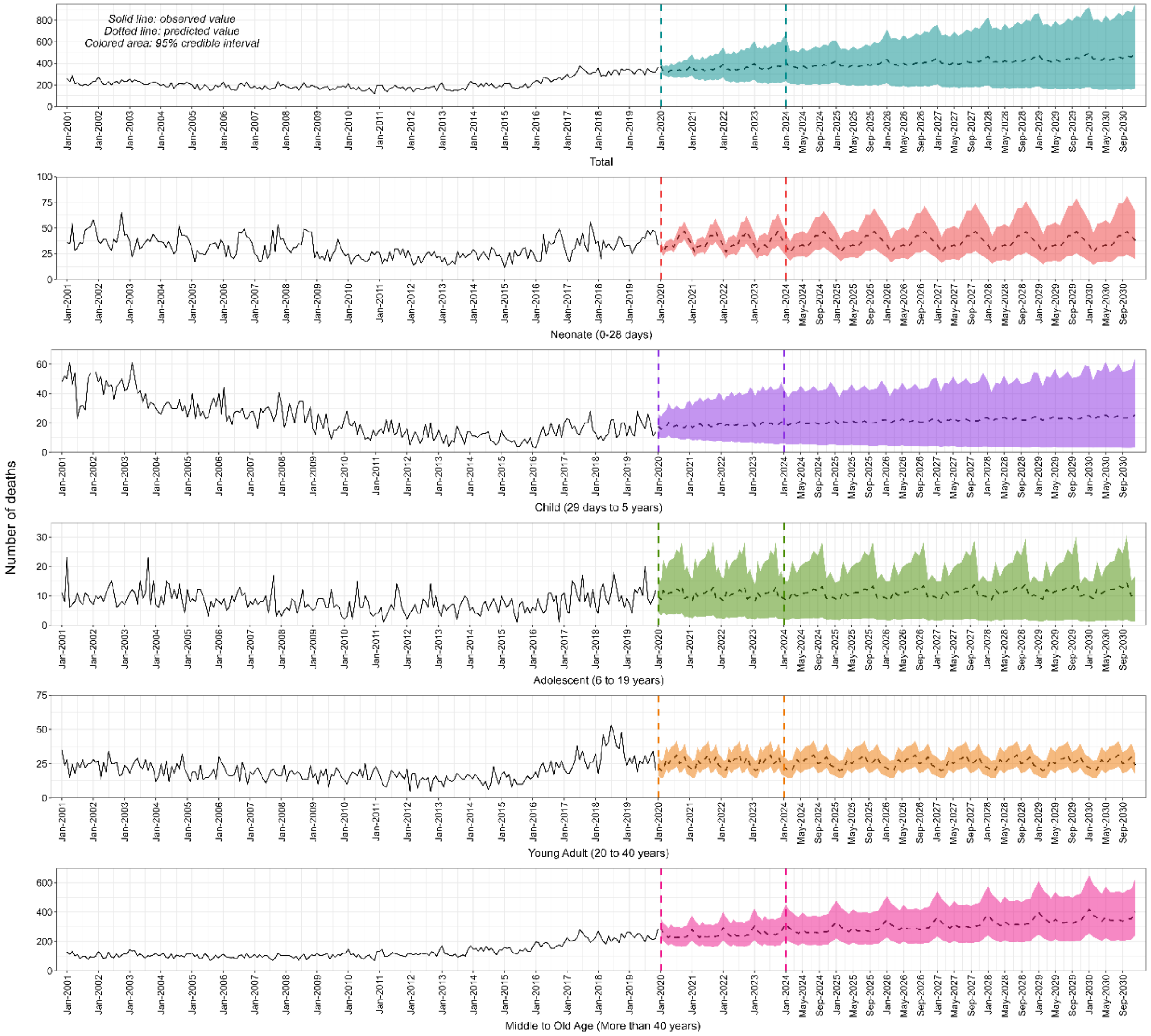

Appendix S1: Adjustment of autocorrelation and seasonality effect in the model

To address the presence of autocorrelation and seasonality in the time-series data, we applied specific adjustments using the INLA (Integrated Nested Laplace Approximation) package in R statistical software. Autocorrelation, which can occur when consecutive observations are correlated over time, was accounted for by incorporating a first-order autoregressive [AR(1)] process in the model using the argument *model = "ar1"*. The AR(1) structure models the current observation as a function of the previous observation, allowing the model to account for temporal dependencies.

In addition to autocorrelation, seasonal variation was modeled by incorporating a seasonal component in the model by specifying the argument *model = "seasonal", season.length = 12*. This adjustment captures the cyclic nature of the data with a frequency of 12, representing monthly seasonality. By setting the season length to 12, the model is able to capture recurring patterns that typically span over a year, such as seasonal fluctuations in deaths. These adjustments, implemented through the INLA framework, ensure that both temporal correlations and seasonal effects are appropriately modeled, leading to more accurate and reliable estimates.
